# Biogenic Synthesis of Fluorescent Carbon Dots (CDs) and Their Application in Bioimaging of Agricultural Crops

**DOI:** 10.3390/nano13010209

**Published:** 2023-01-03

**Authors:** Akshay M. Pete, Pramod U. Ingle, Rajesh W. Raut, Sudhir S. Shende, Mahendra Rai, Tatiana M. Minkina, Vishnu D. Rajput, Valery P. Kalinitchenko, Aniket K. Gade

**Affiliations:** 1 Nanobiotechnology Laboratory, Department of Biotechnology, Sant Gadge Baba Amravati University, Amravati 444602, Maharashtra, India; 2 Department of Botany, The Institute of Science, 15, Madame Cama Road, Mumbai 400032, Maharashtra, India; 3 Academy of Biology and Biotechnology, Southern Federal University, 344090 Rostov-on-Don, Russia; 4 Department of Microbiology, Nicolaus Copernicus University, 87-100 Torun, Poland; 5 All-Russian Research Institute of Phytopathology, 143050 Moscow, Russia; 6 Department of Biological Sciences and Biotechnology, Institute of Chemical Technology, Mumbai 400019, Maharashtra, India

**Keywords:** *Annona squamosa* (L.), carbon dots (CDs), fruit peels, green synthesis, photo luminance

## Abstract

Fluorescent nanoparticles have a transformative potential for advanced sensors and devices for point-of-need diagnostics and bioimaging, bypassing the technical burden of meeting the assay performance requirements. Carbon dots (CDs) are rapidly emerging carbon-based nanomaterials. Regardless of their fate, they will find increasing applications. In this study, a simple approach for synthesizing CDs from fruit peels was developed. The CDs were fabricated from *Annona squamosa* (L.) peels using a carbonization technique through microwave-assisted hydrothermal digestion at temperatures around 200 °C. Synthesized CDs were detected using a UV transilluminator for the preliminary confirmation of the presence of fluorescence. UV–Vis spectrophotometry (absorbance at 505 nm) analysis, zeta potential measurement (−20.8 mV), nanoparticles tracking analysis (NTA) (average size: 15.4 nm and mode size: 9.26 nm), photoluminescence, and Fourier transform infrared (FT-IR) analysis were used to identify the capping functional groups on the CDs. The total quantum yield exhibited was 8.93%, and the field emission scanning electron microscopy (FESEM) showed the size range up to 40 nm. The germinating mung bean (*Vigna radiata* (L.)) seeds were incubated with biogenically synthesized CDs to check the absorption of CDs by them. The fluorescence was observed under a UV-transilluminator in the growing parts of seeds, indicating the absorption of CDs during the germination, development, and growth. These fluorescent CDs could be used as a bioimaging agent. This novel method of synthesizing CDs was found to be eco-friendly, rapid, and cost-effective.

## 1. Introduction

In recent years, carbon nanoparticles (CNPs) have been continuously studied for their photoluminescence properties. Though CNPs cause blue or green photoluminescence, their levels are lower than those of fluorescent carbon quantum dots. Carbon nanomaterials (CNMs) have a peculiar property that enables them to enter a living cell without any further modification or functionalization. This allows their potential application in cell imaging. CNMs showed new, unique, and improved properties like fluorescence, photoluminescence, photon absorption, and scattering because of their higher surface area-to-volume ratio with a decreasing particle size [1,2,3,4]. Nanotechnology has promising applications in diagnostic biomarkers, cell labeling, contrast agents for biological imaging, microbiocidal agents, and a vehicle for drug delivery systems for the treatment of various diseases [5,6,7]. There are several fluorescent nanomaterials (NMs) such as fluorescently doped silica and sol-gels [8], hydrophilic polymers (hydrogels) [9,10], semiconducting polymer dots [11], quantum dots, carbon dots [12], other carbonaceous nanomaterials, hydrophobic organic polymers [13,14], up-conversion NPs [15], various other nanomaterials like noble metal NPs (mainly gold and silver) [16,17,18], and dendrimers [19] that have been reported for their application in bioimaging [20,21]. Other applications of CDs include heavy metal sensing and antimicrobials [22,23]; antibiotic carriers; and fluorescent probes [24]. Despite all of their advantages, they are not without limitations. As there is a limitation in the introduction of specific functional groups, it becomes difficult for an additional coating, water solubility, and fluorescence quenching due to high chromophore loading [20].

Carbon-based materials like carbon black, until recently, were generally considered to be water-soluble [22] and weakly fluorescent. However, organic carbon compounds are sparingly soluble in water [25]. Animal and plant wastes, industrial wastes, etc., were reported for the synthesis of CDs, where the fabrication was based on various transformation processes [26]. Natural organic materials like hair, fruit peels, plastic, etc., are rich in various biomolecules [27,28,29,30]. The higher production of wheat crops can be achieved using water-soluble CDs, which might be an attribute of their growth promotion activity and easy assimilation [31]. Carbon dots (CDs) with excellent optical properties and cytocompatibility are an ideal class of nanomaterials applied in the field of biomedicine. These tiny CDs have recently attracted wide attention globally because of their strong fluorescence, for which they are referred to as fluorescent CDs [32]. CDs are a novel class of zero-dimensional nanoparticles with sizes below 10 nm [33,34,35], first obtained during the purification of single-walled carbon nanotubes (SWCNTs) through electrophoresis in 2004. CDs have gradually become the center of attraction in the carbon family, due to their benign, abundant, and inexpensive nature and their unique properties that reveal a strong fluorescence.

Until recently, a wide range of CDs synthesis and fabrication methods has been reported [36]. The physical methods involve laser ablation, arc discharge, passivation, and a plasma treatment. The biological methods include the synthesis of CDs using bacterial, fungal, plant, and animal extracts as well as by-products. Biological methods have gained more importance because they are rapid, less toxic, cost-effective, and, most importantly, eco-friendly. CDs have inspired intensive research interests in the scientific community in recent times because of their unique properties like facile functionalization, fluorescence, and resistance to photobleaching. Therefore, CDs provide a variety of promising applications in photocatalysis, bioimaging, and optoelectronic devices [37]. The carbon nanotube enhances the roots in onions and cucumbers [38]. The increased reactive oxygen species (ROS) and decreased cell viability were observed in rice plants [39], as well as the growth stimulation of *Arabidopsis* mesophyll cells [40]. Despite their in vitro toxicity in the biological systems and environment, most CNMs have proven to have a huge range of applications in biological systems [12,41]. CDs synthesized and fabricated from various precursors have shown their solubility in polar solvents like water, their assimilation by actively growing plant cells, and the enhancement of the plant growth by an accumulation inside plant tissues and thereby improving the photosynthesis rate [42]. Thus, there is a need to develop an environmentally benign approach for the synthesis of carbon nanoparticles with high photoluminescence properties, and this is still a great challenge. From the available literature, it is observed that little work has been carried out on the biological synthesis of fluorescent CDs and their applications in various fields.

The present study accounts for the fruit peels-based synthesis of CDs, the optimization of the synthesis process, and their application for in-planta photo-imaging in germinating *Vigna radiata* (L.), i.e., mung bean seeds, which could be a novel approach and application in the bioimaging of agricultural crops.

## 2. Materials and Methods

### 2.1. Collection and Preparation of Fruit Peels Powder

Initially, all the fruit peels (*Musa acuminata* (L.), *Citrus limetta* (L.), *Citrus indica* (L.), and *Annona squamosa* (L.)) were collected from local fruit markets in Amravati city, Maharashtra, India. The fruit peels were cleaned by washing them under running tap water separately to remove any dirt residue and other possible sources of contamination. The cleaned peels were washed with distilled water three times to remove any remaining surface impurities. The procedure for the fruit peel primary carbonization was adopted from the reports of Sheth and Patel [43] to obtain the activated carbon powder. The cleaned peels were dried in a hot air oven at 200 °C for 2 h for carbonization [43,44]. The carbonized fruit peels were collected and ground individually with the help of a mixer grinder to make a fine powder, which was then sieved through a muslin cloth to remove any coarse particles. The fine carbonized powders were stored separately in an airtight bag to avoid any contact with the air and humidity.

### 2.2. Optimization of CDs Synthesis and Observation under UV-Transilluminator

The CDs were synthesized from various fruit peels by microwave-assisted hydrothermal digestion. The process parameters were optimized to develop a reproducible method for the CD synthesis, as mentioned. For the optimization of the CDs synthesis, pre-carbonized fruit peel powders were dissolved in distilled water separately (100 mg/25 mL). The CDs were synthesized by the microwave pyrolysis method. Then, they were baked in a microwave oven (LG MC- 7148MS) at various temperatures (160 °C, 320 °C, 480 °C, 640 °C, and 800 °C) and for various time intervals (10, 20, 30, 60, and 150 min), respectively. These baked solutions were filtered through a 0.2 µ filter (TRANSONS ROCKYVAC 400) and centrifuged (Beckman Coulter 64R) at 20,000 rpm for 20 min to remove of any impurities. These solutions were then observed under a UV-transilluminator (Clever Scientific Ltd., Kingston Upon Thames, UK) at 365 nm for the preliminary confirmation of the synthesis of the CDs. The synthesized CDs were primarily detected by observing the visual color change and exposing them under a UV-transilluminator at 365 nm.

### 2.3. Quantum Yield of A. squamosa-Mediated CDs

The quantum yield (QY) of the samples was estimated according to Equation (1), where r refers to the reference and I, A, and n represent the emission intensity, absorbance intensity, and refractive index of the solvent, respectively. Quinine sulfate in 0.1 M of H_2_SO_4_ aqueous solution was taken as the reference. To avoid the inner filter effect, the concentration of the sample aqueous solutions was set at 0.005 mg mL^−1^ [45,46].

(1) QYsample= QYr×[(I sample×A r×nsample2)/(Ir×A sample×nr2)]

### 2.4. Characterization of Carbon Dots (CDs)

#### 2.4.1. UV–Vis Spectrophotometric Analysis

The biosynthesized CDs were detected by a double-beam UV–visible spectrophotometer (Shimadzu UV-1700, Shimadzu Precision Instruments, Inc., Kyoto, Japan). It is one of the well-known and well-established facts that there is a relationship between UV–visible absorbance patterns and the size, shape, and morphology of nanoparticles [3,47,48]. Nanoparticles synthesized by the biological approach showed a broad peak, which indicates the polydisperse nature of the NPs [49].

#### 2.4.2. Nanoparticle Tracking and Analysis (NTA) and Field Emission Scanning Electron Microscopy (FESEM) Studies

The NTA LM 20 is a laser-based light scattering system in which the average particle size and particle size distribution are determined under the LM viewing unit. NTA utilizes both the light scattering and Brownian motion of CDs [50]. Field Emission Scanning Electron Microscopy (FESEM) was performed to measure the exact size of the CDs and visualize their shape by surface scanning under an electron beam of 18.00 kV for 10 µs.

#### 2.4.3. Zeta Potential Analysis

Malvern’s Zeta Sizer (Nano ZS-90, Malvern Panalytical, Malvern, UK) is a high-performance molecular size analyzer used for the measurement of the zeta potential, which is a measure of the charge and stability of the nanoparticles at a pH value of 7. Nanoparticles with a zeta potential more positive than +30 mV or more negative than −30 mV are considered to be stable [51]. The stability of the colloidal systems is indicated by the magnitude of the zeta potential.

#### 2.4.4. Fourier Transform Infrared (FT-IR) Spectrophotometric Analysis

The FT-IR analysis is performed to reveal the presence of various functional groups on a CDs’ surface as a capping agent for the stability of the CDs. The FTIR spectra were recorded using a BRUKER Optics Alpha ATR (Germany) unit for synthesized CDs and peel powder as a control. The scans recorded were an average of 40 scans, and the contribution of the background was accounted for. Each sample was measured in transmission mode at a resolution of 4 cm^−1^ [52,53,54].

#### 2.4.5. XRD and EDX Analysis of Biogenically Synthesized Carbon Dots (CDs)

X-ray diffraction analysis was performed to elucidate the purity and physical nature of the CDs and to determine their purity [55]. Energy Dispersive X-Ray Analysis (EDX), also called Energy Dispersive Spectroscopy (EDS), was made to confirm the elemental composition and presence of any elemental impurities or doped elemental components in the CDs [53,56].

#### 2.4.6. Photo Luminance Analysis

A photo luminance study is performed by a fluorescence spectrophotometer (F-7000), in which the fluorescence emission and excitation were studied. A compact system capable of performing many new functions such as sensitivity (S/N 800: RMS) and ultra-high speed (60,000 nm/min) at the highest level of its class [53,57,58,59].

### 2.5. Evaluation of Effect of CDs on the Germination of Mung Bean (Vigna radiata (L.)) Seeds

Mung bean (*Vigna radiata* (L.)) seeds were purchased from the local market and kept in a dry condition at room temperature before their use. The synthesized CDs were dissolved in water at different concentrations in a 1:1 proportion and were used to evaluate their effect on the germination of mung bean seeds [60,61].

#### 2.5.1. Germination Experiment

The mung bean seeds were first washed with tap water to remove any dust particles or other residues, then with distilled water. Then, the seeds were washed with a 2% Tween 80 solution for 3 min followed by rinsing three times in distilled water. Then, the seeds were sterilized with 0.5% mercuric chloride for 6 min to ensure the surface’s sterility. Again, the seeds were washed with sterilized water three times and later soaked in distilled water (the control) and the CDs’ solution for 6 h, tied in a muslin cloth and placed in moist conditions overnight for germination. Different concentrations of CDs were prepared in Petri plates, and 5 seeds were transferred into each plate and kept in moist conditions at room temperature for 7 days. After the incubation period, the shoot length, root length, and fluorescent emission were measured. Each treatment was conducted in triplicate and repeated several times. The results obtained, i.e., the root length, shoot length, and germination percentage were calculated as the mean ± SD (standard deviation) [60,61].

#### 2.5.2. In-Planta Photoluminescence Detection in Germinating Mung bean (*V. radiata *(L.)) Seeds

The germinating mung bean (*V. radiata *(L.)) seeds were examined for the presence of in-planta photoluminescence after the treatment with CDs in the aqueous phase by observing the seeds under UV light [62].

## 3. Results

### 3.1. Optimization of Carbon Dots (CDs) Synthesis and Detection under UV-Transilluminator

**Figure 1:**
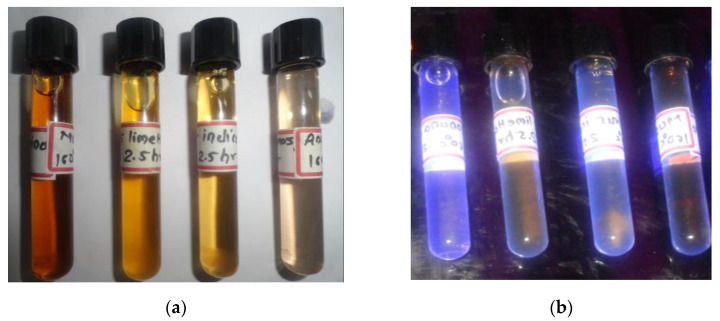
(**a**) Synthesized CDs solutions from different fruit peels from left to right – *Annona squamosa* (L.), *Citrus indica* (L.), *Citrus limetta* (L.), and *Musa acuminata* (L.); (**b**) CDs solutions under UV-transilluminator.

The hydrothermal digestion of the carbonized solution from all of the selected fruit peel was used to create CDs, which were then placed in transparent screw cap bottles for the detection of their fluorescence under a UV-transilluminator (Figure 1a,b). Among the selected filtrates, *A. squamosa* (L.) digested at 160 °C for 150 min showed a better fluorescence compared to the others. Therefore, the CDs prepared from *A. squamosa* (L.) peels were selected, characterized, and used for a further testing. As a medium for the CDs synthesis, water results in less yield (83 mg per 1 g of digested fruit peel carbonized powder; nearly 8%).

### 3.2. Characterization of Carbon Dots (CDs)

#### 3.2.1. Quantification of Carbon Dots (CDs)

The quantum yield (QY) of the CDs synthesized for the hydrothermal carbonization of *A. squamosa* peel was calculated based on the PL intensity and absorbance. In the present study, *A. squamosa*-mediated carbon dots exhibited a QY of 8.93%, which was probably due to the presence of nitrogen containing functional groups in the CDs.

#### 3.2.2. UV–Vis Spectrophotometric and Photo Luminance Analysis

**Figure 2:**
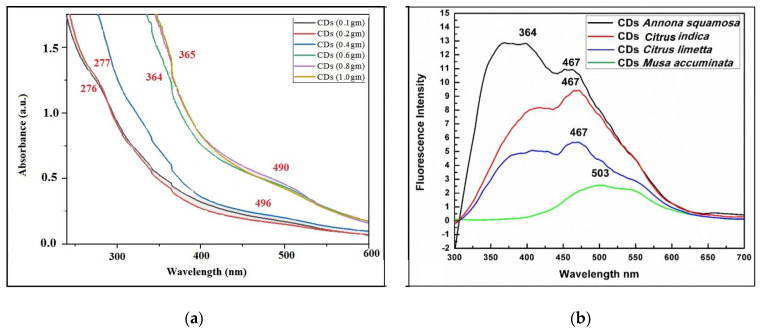
(**a**) UV–Vis spectrum of *Annona squamosa*-mediated CDs synthesized at various concentrations; (**b**) photoelectric luminance analysis (excitation wavelength 364 nm).

A spectrophotometric and photoluminescence analysis was conducted to determine the absorbance maxima in the UV–Vis range, as well as their emission and excitation wavelengths. Various concentrations of *A. squamosa* peel powder (0.1, 0.2, 0.4, 0.6, 0.8, and 1.0 g per 25 mL of distilled water) showed different absorbance maxima at 276 nm, 277 nm, 496 nm, and 490 nm, respectively (Figure 2a). The observed deflections in the spectrum are likely caused by electron π−π* transitions. For *A. squamosa*, the aqueous CDs solution exhibited the strongest photoluminescence at 467 nm (Figure 2b). The CDs synthesized by *A. squamosa* peels were selected for a further study after the UV–Vis spectrum and fluorescence emission of different plant samples. It was found that the excitation wavelength for the CDs was between 270 nm and 370 nm, and the fluorescence peak for the CDs was between 420 nm and 500 nm.

#### 3.2.3. Nanoparticles Tracking and Analysis (NTA)

**Figure 3:**
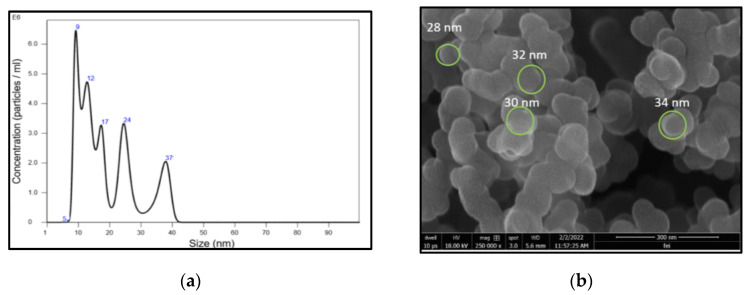
(**a**) NTA (Nanosight-LM-20) nanoparticle size distribution histogram showing the average size of 15.4 nm; (**b**) FESEM image of CDs at 250,000× resolution.

To determine the size and concentration of synthesized CDs, NTA was conducted. Based on the NTA, the average CD diameter was 15.4 nm, with a mode of 9.26 nm and a standard deviation of 11.4 nm. Figure 3a shows the synthesis of *A. squamosa* CDs with diameters ranging from 5 to 37 nm. In the sample examined, the CDs accounted for 6.39 × 10^8^ particles mL^−1^. The FESEM depicted the spherical shape of CDs, with their sizes ranging below 40 nm. The FESEM image of the CDs at 18 kV and the 300 nm scale with a resolution of 250,000× is shown in Figure 3b.

#### 3.2.4. Zeta Potential Analysis

**Figure 4:**
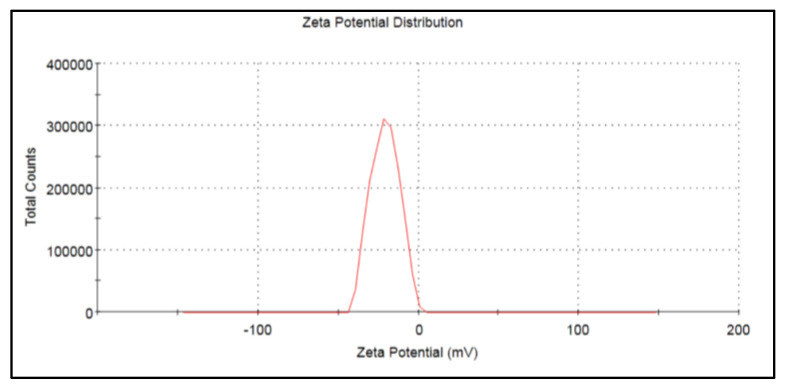
The zeta potential graph for biosynthesized CDs shows a zeta potential at −20.8 mV.

The zeta potential measurements were carried out to determine the stability of CDs in a colloidal or soluble form. The zeta potential was found to be −20.8 mV (Figure 4), which indicates that the nanoparticles were moderately stable.

#### 3.2.5. Fourier Transform Infrared (FT-IR) Spectrophotometric Analysis

**Figure 5:**
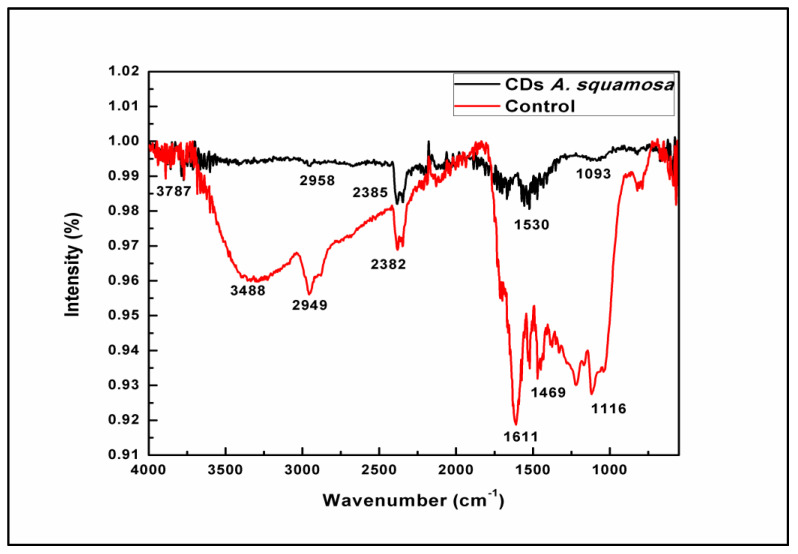
Fourier transform infrared (FTIR) spectral analysis of *A. squamosa* CDs.

The FT-IR analysis was performed to determine the biochemical composition and exposed functional groups present in the CDs. A main characteristic absorption band of the O-H and N-H stretching vibration modes at 3787 cm^−1^ was observed, while 2958 cm^−1^ gives a C-H and S-H stretching vibration mode. The band at 2385 cm^−1^ corresponds to polycyclic aromatic hydrocarbons stretching into C=C molecules. N-H bending vibrations at 1643 cm^−1^ and the band at 1093 cm^−1^ correspond to C-O-C and C-H bending vibrations of the pyranose ring in fruit peels, which may have assisted in the capping and stabilization of CDs (Figure 5).

#### 3.2.6. XRD and EDX Analysis of Synthesized CDs

**Figure 6:**
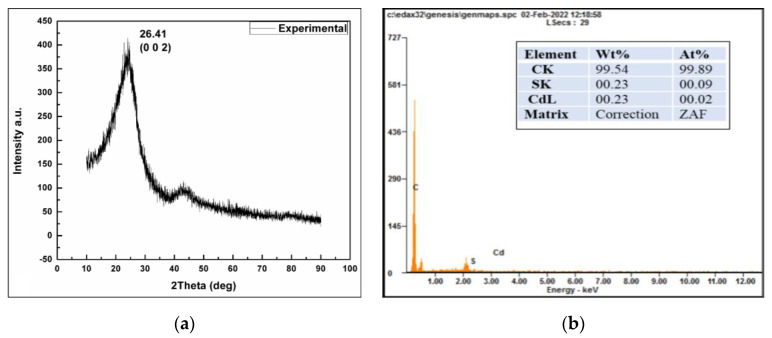
(**a**) X-ray diffraction pattern; (**b**) EDX spectrum of *A. squamosa* CDs.

The XRD pattern of the CDs shows a broad peak at 26.41° corresponding to the (002) *hkl* plane (JCPDS card no. 26-1076) as per Figure 6a. Energy-dispersive X-ray (EDX) spectroscopy depicted 99.54 % of the carbon content with minor signs of cadmium and sulfur impurities, as shown in Figure 6b.

### 3.3. Evaluation of the Effect of CDs on Germination of Mung Bean (Vigna radiata (L.)) Seeds

#### 3.3.1. Germination Experiment

**Figure 7:**
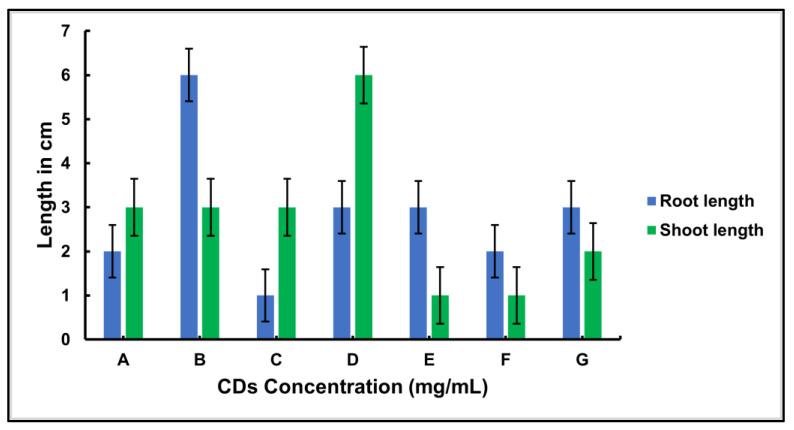
Effect of fluorescent CDs on Germinating seeds of Mung bean (*V. radiata* (L.)) after 8 Days of incubation. Error bars indicate the uncertainty or errors in the measurements reported. Where A, B, C, D, E, F, and G refer to the treatment of germinating seeds with CDs at concentrations of 1, 2, 4, 8, 12, 16 mg/mL, and untreated (the control) seeds, respectively.

The CDs were tested for their ability as growth enhancers or inhibitors on sprouted seeds, their in-planta fluorescence in germinating seeds, and their phytotoxicity. After surface cleaning, the mung bean seeds were treated with various concentrations of CDs prepared from *A*. *squamosa* (L.). The germination of the seeds was monitored for the growth of the root and shoot at room temperature. Figure 7 shows the effect of CDs on both seeds after 8 days of incubation. During the 8 days of incubation, it was observed that the root length in the mung bean seedlings reached its maximum at a concentration of 4 mg/mL and the shoot length was highest at a concentration of 16 mg/mL.

#### 3.3.2. In-planta Photoluminescence Detection

**Figure 8:**
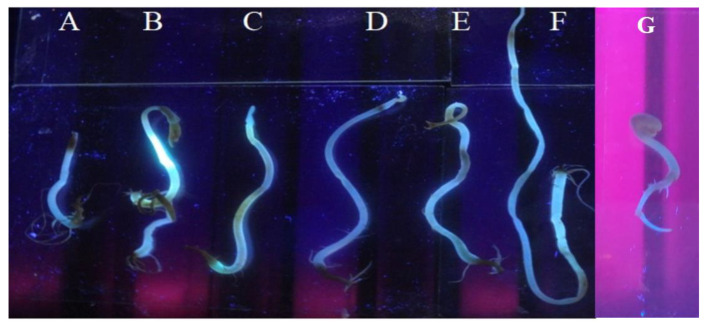
In-planta effect of CDs on *V. radiata* (L.) seeds; fluorescence in germinating seeds due to absorption of CDs; where (**A**–**G**) refer the treatments of CDs on germinating seeds at concentrations of 1, 2, 4, 8, 12, 16 mg/mL, and untreated (the control) seeds.

The assimilation of CDs by the plant cells was determined by detecting the photoluminescence in the treated germinating seeds. When observed under UV-light (302/312 nm (UV-B)), the germinated mung bean seeds showed a higher level of light emissions, and the effect was not dose-dependent (Figure 8). Further, no functionalization of the synthesized CDs was required to be absorbed in the shoot cells of *V. radiata* (L.). The above excitation and fluorescence analysis (Figure 2) led us to conclude that CDs emit a green or blue fluorescence when exposed to UV light. Figure 8 shows the blue-green fluorescence caused by the CDs-treated seeds and growing parts, which were confirmed to have absorbed CDs without being functionalized. In the absence of CDs, no fluorescence was observed in the control seeds (Figure 8G).

## 4. Discussion

Carbon Dots (CDs) offer a great potential for a broad range of applications such as energy-saving, bioimaging, biosensors, lasers, and light-emitting diodes [4,35,63,64]. They may also be applied as a promising new type of fluorescence marker as well as a high-efficiency catalyst design for applications in bioscience and energy technology [4,65,66]. In a study, Das and colleagues reported a one-step synthesis of fluorescent CDs for biolabeling [67]. A simple and facile one-pot synthesis of the fluorescent CDs from orange waste peels was performed using the hydrothermal carbonization method at a mild temperature (180 °C). Likewise, in the present study, the biogenic synthesis of the CDs in the aqueous system results in a lesser yield, as mentioned in the results Section 3.1, i.e., 83 mg per 1 g equating to nearly 8%, however, is more economical than other organic solvents like PEG (polyethylene glycol) used in the synthesis of CDs reported earlier in several studies [23,32,35,61,68]. The QY analysis demonstrated a lesser QY, that might be an attribute of larger CDs and a broad size distribution. The presence of nitrogenous compounds contributing towards the stability of CDs was confirmed with the FT-IR analysis in Section 2.4.4. The presence of N-H stretches, and bends was confirmed in the FT-IR spectrum. Other reported studies on the quantum yield of the CDs derived from various precursors showed a vast range of QY from 7.8% [45] to 85% [23,46,58].

A spectrophotometric and photoluminescence analysis showed a different absorbance maxima at 276 nm, 277 nm, 496 nm, and 490 nm, which are in line with the previous studies that also described the deflections in the spectrum caused due to the electron transitions at π−π* [69,70]. The morphological features and chemical composition of the obtained CDs were evaluated using various spectroscopies and transmission electron microscopy (TEM). The carbonization and functionalization occur through the dehydration of the orange peels, leading to the formation of nano-sized fluorescent carbon particles [71]. It was also found that the excitation wavelength for the CDs was between 270 nm and 370 nm, and the fluorescence peak for the CDs was between 420 nm and 500 nm. This indicated the emission of a blue or green fluorescence [23,72,73]. In the NTA, we observed individual nanoparticles in motion and their size distribution, which could be used to estimate the number of particles mL^−1^ in the solution. The NTA results corroborate the TEM analysis of the C-dots synthesized from orange waste peels [63].

The negative zeta potential value of the nanoparticles may be attributed to the negatively charged functional groups present on the surface of the CDs created from fruit peel [51,74,75,76]. In the study of Wu et al. [77], the observed zeta potential values were consistent with the reports of a stable CD synthesis [35]. The FT-IR analysis showed the peaks depicting the functional groups capped and stabilized the CDs. Prasannan and Imae [63] reported that CDs exhibit a main characteristic absorption band of O-H and N-H stretching vibration modes at 3787 cm^−1^, while 2958 cm^−1^ gives a C-H and S-H stretching vibration mode. The band at 2385 cm^−1^ corresponds to polycyclic aromatic hydrocarbons stretching into C=C molecules. The N-H bending vibrations at 1643 cm^−1^ and band at 1093 cm^−1^ correspond to C-O-C and C-H bending vibrations of the pyranose ring in fruit peels, which may have assisted in the capping and stabilization of the CDs, as shown in Figure 5 [45,57,63].

The XRD pattern revealed a broad peak at 26.41° when compared with JCPDS card no. 26-1076; it was analogous to the plane (*hkl*), corresponding to the Miller index (002) for the CDs. This indicates that the space between the layers is larger than the graphitic spaces (0.33 nm), as is the width. As such, C-dots have a poor crystalline structure [55]. The EDX spectroscopy showed 99.54% of the carbon content with minor signs of cadmium and sulfur impurities that corroborated the report by Hu et al. [56].

After the characterization as an application part for the bioimaging, the CDs were tested for their ability as growth enhancers or inhibitors of sprouted seeds, their in-planta fluorescence in germinating seeds, and their phytotoxicity. The mung bean seeds were treated with the CDs synthesized by *A. squamosa* (L.). A significant effect of the CDs on the root and shoot growth and development was observed after 8 days. At a concentration of 4 mg/mL CDs, the root growth was at the maximum while the shoot length was highest at the concentration of 16 mg/mL. It was found that other types of carbon nanomaterials are toxic to plants, such as multi-walled CNTs and fullerenes demonstrated toxic effects on corn (*Zea mays* (L.)) and soybean (*Glycine max* (L.)), which reduced the biomass [78]. The above results indicated that CDs produce a dose-dependent effect, depending upon the growth stage. In plants, the growth promotion may depend on the ability of cells to assimilate the nutrients, which may vary with the developmental stages [61,70,79]. The CDs’ specific optical and chemical properties make them reasonably hydrophilic and ease their transport across plant membranes [80]. The present study indicates the promotion of seed germination and growth due to a certain concentration of CDs. Higher concentrations might be responsible for having no further effect on the germinating seeds. This indicates the dose dependency of the developing seeds on the CDs during the crucial growth stages of seed germination in the case of onions and watermelons [81,82]. The plant physiology is altered to a certain extent to obtain the desired variation in the plant system [42,83].

Chemical and physical methods are more effective. Wang and Hu [84] summarize the advantages and disadvantages of various approaches to CDs synthesis. There has been some difficulty in the synthesis and application of major physical and chemical methods. The method reported here is facile, reproducible, quick, and generates CDs of a range of sizes and can also be used to design bioassays based on their fluorescence properties. Additionally, the in-planta results reported here support the application of *A. squamosa*-based CDs in plant bioassays and in vivo biolabeling [68,78]. These biocompatible CDs can thus be assessed for their fate in vivo and be applied in biolabeling studies to understand the various biomolecular pathways and predict the desired spatial distribution of the nanomaterials [4,35,85,86].

## 5. Conclusions

The current use of bioimaging dyes and agents are mostly chemicals, which are hazardous to humans and the environment. Chemical dyes damage the cell wall, are more expensive, and can only stain dead cells. In the present study, a green, eco-friendly, and economically viable approach has been developed for the synthesis of CDs. CDs synthesized from plant-based materials are eco-friendly and cost-effective compared to chemical agents. CDs were assimilated by the cells of germinating mung bean (*Vigna radiata* (L.)) seeds without the further functionalization for their entry into plant tissues. CDs also showed a significant effect on the seed germination in mung beans. It is suggested that the fluorescent CDs synthesized from fruit peels by the microwave-assisted method could be used as a bioimaging agent because there were no signs of any visible phytotoxic effect on the germinating seeds, as indicated by zero mortality at any concentration used for the assessment; thus, indicating their biocompatibility with the plant systems for the development of biolabeling assays.

Moreover, the findings of the present study would be useful in understanding and addressing the problem of toxicity associated with chemical bioimaging dyes. Further, thorough studies on the conjugation of CDs with the desired biocatalyst for specific functions in-planta would be helpful to reveal the applicability of CDs in various fields. The present findings open a new avenue for the commercialization of CDs as a viable alternative to synthetic organic dyes as multi-color emitting probes for cell labeling in bioimaging.

## Data Availability

Not applicable.
